# Unusual Presentation of Renal Medullary Carcinoma With Undiagnosed Sickle Cell Trait

**DOI:** 10.7759/cureus.10731

**Published:** 2020-09-30

**Authors:** Fahmin Basher, Giselle Dutcher, Jonathan S England, Gilberto Lopes

**Affiliations:** 1 Division of General Internal Medicine, Department of Medicine, University of Miami Miller School of Medicine, Jackson Memorial Hospital, Miami, USA; 2 Divisions of Hematology and Medical Oncology, Department of Medicine, University of Miami Miller School of Medicine, Jackson Memorial Hospital, Miami, USA; 3 Department of Pathology, University of Miami Miller School of Medicine, Jackson Memorial Hospital, Miami, USA; 4 Division of Medical Oncology, Department of Medicine, University of Miami Miller School of Medicine, Jackson Memorial Hospital, Miami, USA

**Keywords:** renal medullary carcinoma, renal cell carcinoma, sickle cell trait

## Abstract

Renal medullary carcinoma (RMC) is an extremely rare malignancy that has been described in younger male patients of African descent with a history of sickle cell disease or trait. We describe a rather unique case of RMC in an older male patient who initially presented with acute on chronic urinary retention and concern for infection. Further investigation revealed a history of hematuria and long-standing microcytic anemia, and the patient was found to have sickle cell trait (SCT) as part of a workup for malignancy of unknown primary. Imaging findings initially interpreted as hydronephrosis later characterized a mass in the renal pelvis concerning for a genitourinary malignancy, later biopsy-proven RMC. RMC typically presents in its advanced stages, with associated poor prognosis, and treatment options are limited and have been extrapolated from standard regimens for other genitourinary malignancies. Therefore, early clinical suspicion in patients with microcytic anemia, flank pain, hematuria, and urinary symptoms, can aid in the diagnosis of RMC and allow for prompt intervention.

## Introduction

Renal medullary carcinoma (RMC) is a rare malignancy first described in 1995 as the “seventh sickle cell nephropathy,” with nearly 90% of cases reported to date associated with sickle cell trait (SCT) [1]. RMC was initially observed in a population of 34 patients, nearly all African-American, and predominantly young males, with known hemoglobinopathies (either sickle cell trait or hemoglobin SC disease) [2]. The initial clinical presentation typically includes hematuria and/or flank pain with concern for urinary tract infections or abscesses, with suspicion for malignancy only increased after the failure of antibiotic therapy [3,4]. Additionally, patients may describe fatigue and weight loss, and symptoms are present on average about five months prior to diagnosis [1]. Therefore, it is not unusual that patients at the time of presentation exhibit metastatic disease with the most commonly described sites of metastasis being liver, bone, and adrenal glands, as well as regional lymphadenopathy [5]. We present a rather unusual case of RMC in an older adult male with undiagnosed sickle cell trait and a clinical presentation of acute urinary retention and pyelonephritis with widespread suspected metastatic disease on imaging.

## Case presentation

Our patient is a 62-year-old Honduran male who initially presented to the urology clinic for a scheduled urodynamic study for evaluation of recurrent acute urinary retention accompanied by fever, chills, and suprapubic discomfort. His known medical history included benign prostatic hypertrophy, and his surgical history included exploratory surgery at the age of 20 in Honduras for hematuria. The patient was noted to be febrile and was sent to the emergency room. Labs on presentation were notable for microcytic anemia (hemoglobin 7 g/dl, normal 13-16 g/dl, with mean corpuscular volume of 70 fL, normal 80-95 fL), leukocytosis (29.8 x 10^3^/µL, normal 4.0-10.5 x 10^3^/µL), elevated lactic acid (3.7 mmol/L, normal 0.7-2.1 mmol/L) and urinalysis notable for pyuria. He was admitted for intravenous antibiotics for pyelonephritis. 

Computed tomography (CT) scans and magnetic resonance imaging (MRI) of the head, chest, abdomen, and pelvis were performed that revealed extensive multi-organ involvement, including diffuse lymphadenopathy and bony disease. In particular, MRI of the brain displayed T2 hyperintense lesions in the left mandibular condyle, measuring 2.6 cm with cortical erosion into the infratemporal fossa, and left masseter muscle (measuring 1 cm) as well as in the left parotid gland (measuring 1.1 cm). Chest imaging demonstrated multiple prominent mediastinal and hilar lymph nodes, as well as innumerable solid noncalcified pulmonary nodules throughout both right and left lungs, measuring 0.3-0.6 cm. CT also demonstrated numerous sclerotic lesions in the cervical and thoracic spine, particularly at the levels of C7, T4, and T10, as well as in the left clavicle and left humerus and a pathologic fracture in the left fourth rib. Dedicated bone imaging with positron emission tomography (PET)-CT also demonstrated diffuse uptake throughout the axial and appendicular skeletons, including but not limited to the skull bone, mandible, clavicles, shoulders, multiple ribs, cervicothoracic spine, bony pelvis, and proximal femurs.

**Figure 1:**
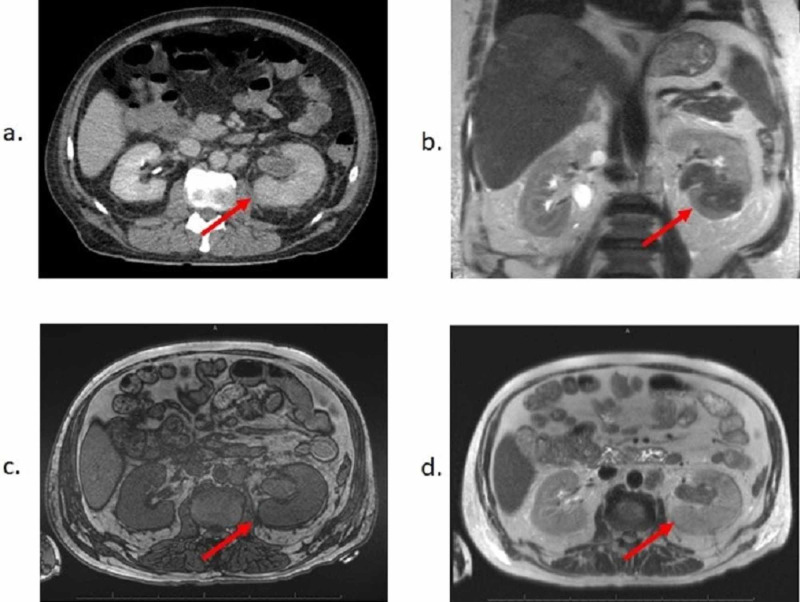
Initial Imaging of Renal Pelvis Mass. Representative images from (a) CT pelvis with contrast, (b) MRI pelvis scout, (c) T1-weighted MRI, and (d) T2-weighted MRI demonstrating lesion occupying left renal pelvis with decreased enhancement and delayed excretion of contrast, with adjacent soft tissue stranding. Mass in left kidney is restricting and extends into the renal parenchyma into the lower pole of left kidney, measuring up to 7 cm in greatest diameter.

MRI and CT imaging also demonstrated considerable soft tissue and solid organ involvement. Notably, the left kidney was noted to be enlarged with decreased enhancement and delayed excretion of contrast, with associated mild hydronephrosis and inflammatory changes on CT (Figure 1a). However, on subsequent MRI, the left kidney was visualized as containing a restricting mass occupying the left renal pelvis, extending into the lower pole of the left kidney, measuring 7 cm in greatest diameter, with an additional T2 bright / T1 dark lower pole lesion measuring 1.9 cm and associated right para-aortic lymphadenopathy measuring up to 2 cm. MRI also demonstrated an exophytic upper pole lesion in the right kidney, also T2 bright / T1 dark without associated hydronephrosis (Figures 1b-1d). Of note, a CT abdomen/pelvis one year prior to this presentation showed bilateral renal cysts and a small indeterminate heterogeneous 1.2 cm lesion in the left kidney. Additional imaging findings suggestive of metastatic disease included multiple hypodense lesions visualized in the liver (measuring 1.5-2.3 cm), a lobulated 3.5 cm hypoenhancing mass identified in the right adrenal gland (with minimal dropout with out-of-phase imaging on MRI), and a left psoas metastasis at the level of L3.

**Figure 2:**
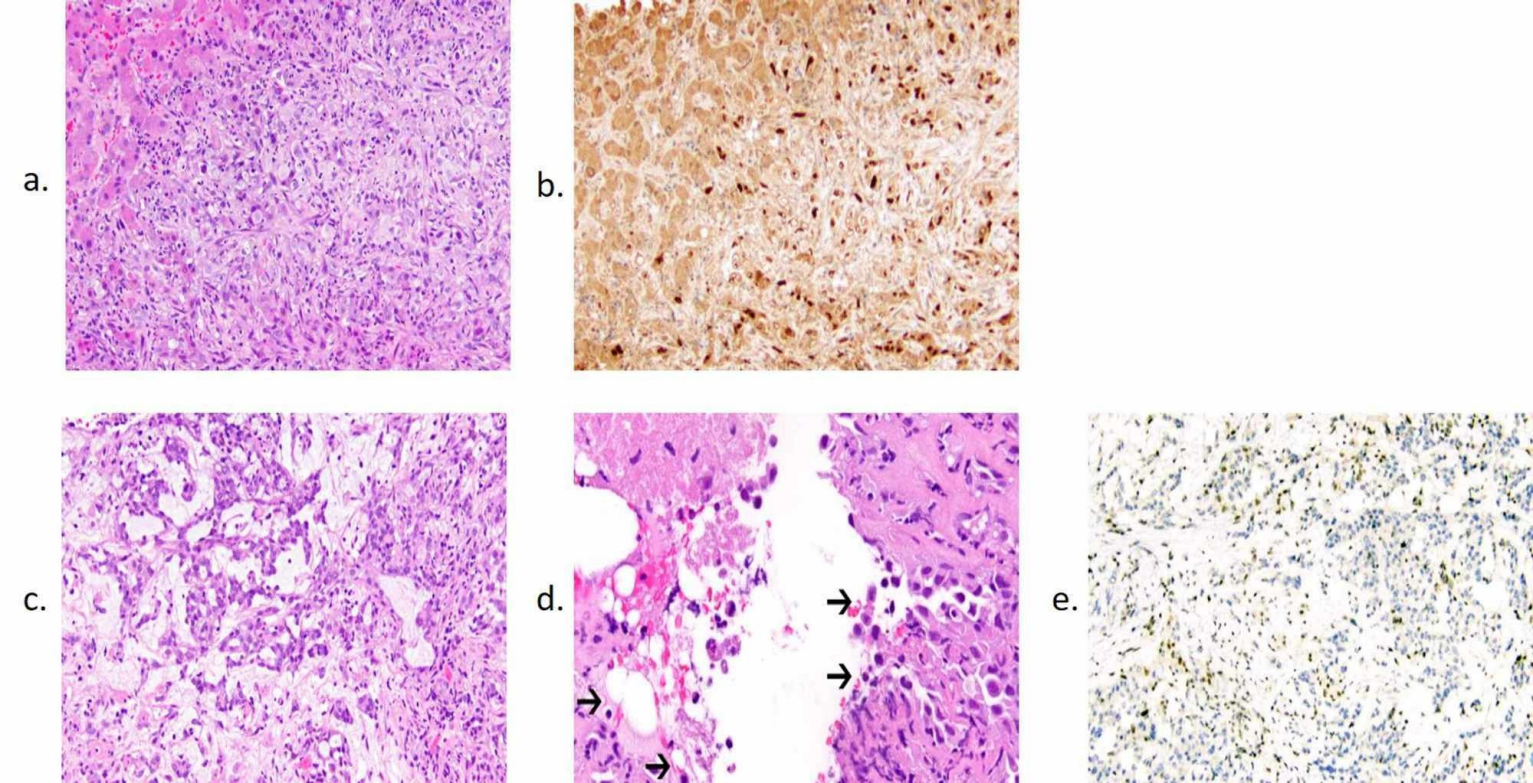
Pathology Specimens of the Liver and Kidney. Hematoxylin and eosin (H&E) staining (a) and immunohistochemical (IHC) staining for PAX8 (b) of the liver lesion specimen shows a PAX8+ poorly differentiated adenocarcinoma. (c) H&E staining of the kidney specimen at 10x magnification shows a high-grade carcinoma with a reticular architectural pattern and prominent desmoplasia. (d) H&E staining of the kidney specimen at 20x magnification demonstrates scattered sickled red blood cells (arrows), characteristic histologic findings of renal medullary carcinoma. (e) IHC for INI1 of the kidney specimen shows aberrant loss of staining in the tumor cells with retention in the background of non-neoplastic cells.

Due to imaging concerning for metastatic disease, the patient underwent a liver biopsy, which showed poorly differentiated adenocarcinoma. Immunohistochemistry (IHC) was largely unrevealing for likely primary aside from positive PAX8 staining, commonly seen in renal, thyroid, and gynecologic malignancies (Figures 2a, 2b). He underwent esophagogastroduodenoscopy and colonoscopy, which were only remarkable for a tubular adenoma in the rectosigmoid colon, and a recent prostate biopsy two months prior to presentation was negative. Hemoglobin electrophoresis was also performed due to microcytic anemia and confirmed sickle cell trait (SCT) with HgbAS.

Upon further review of the initial CT pelvis, which had described hydronephrosis, it was determined that a mass could not be excluded given the location. The patient underwent an MRI, which showed a restricting left renal mass occupying the pelvis and extending into the lower pole of the left kidney at the site previously thought to represent hydronephrosis (Figures 1b-1d). Due to these imaging findings, IHC repeated on previous liver biopsy sample demonstrated loss of expression of INI1/SMARCB1 (integrase interactor 1/SWI/SNF-related matrix-associated actin-dependent regulator of chromatin subfamily B member 1), a known tumor suppressor gene located on chromosome 22q11.2. Subsequent biopsy of the renal mass confirmed RMC, with hematoxylin and eosin (H&E) staining demonstrating a reticular pattern associated with high-grade carcinoma (Figure 2c) as well as the presence of sickled red blood cells (Figure 2d). Notably, IHC of the renal biopsy showed aberrant loss of INI1/SMARCB1 expression (Figure 2e), similar to that seen in the previous liver biopsy. The patient elected to forgo chemotherapy and transitioned to palliative care.

## Discussion

RMC is a rare, aggressive malignancy, representing less than 1% of renal cancers. The median age at diagnosis is 24.3 years and affects males predominantly in a 2:1 ratio [1,3]. Grossly, the tumor is typically a poorly circumscribed mass noted to occupy the renal medulla and often infiltrates into the renal pelvis, and the composition can include cystic lesions, necrotic tissue, and/or hemorrhage [3]. Approximately 70% of these tumors are found in the right kidney, likely due to the longer right renal artery, which reduces effective blood flow to the right renal medulla and increases the likelihood of microinfarctions. However, hematuria in patients with SCT can arise from the left kidney due to an anatomical variant termed nutcracker syndrome, in which compression of the left renal vein by the aorta and superior mesenteric artery results in renal papillary necrosis [6].

The histopathologic analysis may demonstrate variable patterns, but most tumors are found to have reticular, cribriform, and cystic patterns, often with areas of poorly differentiated cells [2]. Nearly all tumors show evidence of acute inflammation with lymphoplasmacytic infiltrate and microabscesses, as well as desmoplastic stromal reaction [5]. Differential diagnoses of genitourinary malignancies include collecting duct carcinomas, which present in older adults and exhibit tubular patterns as opposed to the reticular patterns associated with RMC but share desmoplastic reactions with lymphocytic infiltrate, and urothelial carcinomas, which lack PAX8 staining but retain intact INI/SMARCB1 staining and also present in an older population [7]. Another malignancy to consider as a differential diagnosis is rhabdoid tumor of the kidney, which shares the lack of INI1/SMARCB1, typically secondary to deletions and/or mutations without concurrent translocations, but usually presents in younger children [8]. Other rare variants of renal cell carcinomas (RCC), including fumarate hydratase-deficient RCC and anaplastic lymphoma kinase (ALK)-rearrangement RCC, should be considered due to the molecular pathways involved that render specific therapeutic agents more effective.

The hallmark immunohistochemical finding in RMC is the complete loss of the tumor suppressor gene INI1/SMARCB1, which leads to the upregulation of cyclin D1 and progression through the cell cycle. Fluorescence in situ hybridization demonstrates that this loss of function results from chromosomal translocations and rearrangement in patients with sickle cell disease, with homozygous deletion seen in one patient without known hemoglobinopathy, but no identified de novo mutations of INI1/SMARCB1 [5,9,10]. In fact, INI1/SMARCB1 mutations were first identified in malignant rhabdoid tumors, and the occurrence of balanced translocations in patients with underlying hemoglobinopathies serves as a “second hit” contributing to the pathogenesis of RMC [11].

Given the strong association of RMC with hemoglobinopathies involving sickling, it is reasonable to investigate specific contributions of the renal medullary environment that account for the pathogenesis of this rare malignancy. For the purposes of concentrating urine, the inner medulla is extremely hypoxic and hypertonic, leading to the sickling of red blood cells, even in patients with sickle cell trait, as seen in our patient, though peripheral sickling and its consequences are not clinically apparent. This hypoxic environment is in contrast to cortical areas, where conventional RCCs tend to arise, and triggers the release of pro-angiogenic factors such as hypoxia-inducible factor, tumor protein 53, and vascular endothelial growth factor (VEGF) [12]. Sickling in the renal medulla leads to microinfarctions, exacerbating the innate hypoxia, and further sickling. Concurrently, the hypertonicity leads to an increased incidence of DNA damage as well as suppression of repair responses normally triggered by decreased osmolarity [13].

Treatment options for RMC depend on the extent of the disease and are largely extrapolated from other malignancies of the genitourinary system. Patients with localized disease typically undergo either partial or radical nephrectomy, often with lymphadenectomy, but as most patients present with metastatic disease, nephrectomy is recommended in only about 5% of cases [14]. Prognosis is particularly poor in patients with metastatic disease at the time of diagnosis (four to five months) and median survival of 17 months in patients with localized disease. Patients undergoing nephrectomy had a longer overall survival (six months vs. three months), taking into consideration a selection bias for patients without large disease burden and good performance status [15].

A variety of systemic therapies have been used for patients with RMC, the most common being cytotoxic platinum-based regimens, eliciting an objective response in 29% of patients [16]. Other therapeutic avenues reported to be used in RMC patients with varying degrees of response include topoisomerase II inhibitors, proteasome inhibitors such as bortezomib, anti-angiogenic therapy including bevacizumab, enhancer of zeste homolog (EZH) inhibitors, VEGF inhibitors including sunitinib, and mammalian target of rapamycin (mTOR) inhibitors such as everolimus [5,14]. A newer therapeutic option currently being explored is the class of checkpoint inhibitors including ipilimumab, targeting cytotoxic T-lymphocyte-associated protein 4 (CTLA-4), and programmed death 1 (PD-1) pathway inhibitors nivolumab and pembrolizumab, to potentially target the lymphocytic infiltrate characteristically seen in histologic analysis [14,17].

## Conclusions

In conclusion, RMC should be considered as a differential diagnosis in patients under 50 years of age with known or suspected hemoglobinopathies presenting with classic signs of renal malignancy (hematuria and flank pain) as well as other signs of nephropathy and urinary tract obstruction. Our case highlights an unusual presentation of this rare disease, who presented at a much later age than most RMC patients and also exhibited a long-standing history of urologic complaints without adequate explanation. In addition, our patient’s presentation included a previously undiagnosed sickle cell hemoglobinopathy, which has largely been regarded as benign but likely contributed to the pathogenesis of this malignancy. While prognosis remains relatively poor for advanced RMC, and optimal therapy has yet to be determined, early recognition of the clinical presentation can facilitate more robust treatment responses and bolster awareness of this rare but aggressive malignancy.
